# Purification and Biochemical Characterization of Polyphenol Oxidase Extracted from Wheat Bran (*Wan grano*)

**DOI:** 10.3390/molecules29061334

**Published:** 2024-03-17

**Authors:** Kun Yu, Wei He, Xiaoli Ma, Qi Zhang, Chunxu Chen, Peiyan Li, Di Wu

**Affiliations:** School of Food Engineering, Anhui Science and Technology University, Chuzhou 233100, China; 18244791545@139.com (W.H.); yjs2022285@ahstu.edu.cn (X.M.); 18255213932@163.com (Q.Z.); ccx1205@126.com (C.C.); lipy@ahstu.edu.cn (P.L.); wudi@ahstu.edu.cn (D.W.)

**Keywords:** wheat polyphenol oxidase, purification, biochemical characterization

## Abstract

Currently, little is known about the characteristics of polyphenol oxidase from wheat bran, which is closely linked to the browning of wheat product. The wheat PPO was purified by ammonium sulfate precipitation, DEAE-Sepharose ion-exchange column, and Superdex G-75 chromatography column. Purified wheat PPO activity was 11.05-fold higher, its specific activity was 1365.12 U/mg, and its yield was 8.46%. SDS-PAGE showed that the molecular weight of wheat PPO was approximately 21 kDa. Its optimal pH and temperature were 6.5 and 35 °C for catechol as substrate, respectively. Twelve phenolic substrates from wheat and green tea were used for analyzing the substrate specificity. Wheat PPO showed the highest affinity to catechol due to its maximum V_max_ (517.55 U·mL^−1^·min^−1^) and low K_m_ (6.36 mM) values. Docking analysis revealed strong affinities between catechol, gallic acid, EGCG, and EC with binding energies of −5.28 kcal/mol, −4.65 kcal/mol, −4.21 kcal/mol, and −5.62 kcal/mol, respectively, for PPO. Sodium sulfite, ascorbic acid, and sodium bisulfite dramatically inhibited wheat PPO activity. Cu^2+^ and Ca^2+^ at 10 mM were considered potent activators and inhibitors for wheat PPO, respectively. This report provides a theoretical basis for controlling the enzymatic browning of wheat products fortified with green tea.

## 1. Introduction

Wheat polyphenol oxidase (PPO) is a key factor causing browning in Asian noodles and other wheat products. Consumer acceptability of specialty noodles (such as green tea, vegetable, and whole-wheat noodles) prepared from wheat flour heavily depends on the end-product color. Although wheat grains after milling retain 3% of their total PPO activity [1], the residual PPO is sufficient to cause browning of raw wheat noodles. Particularly, wheat bran with a high PPO content has been widely used for enriching foods with dietary fiber and antioxidants with increasing attention to whole grain foods. The high PPO content in wheat bran products makes them susceptible to enzymatic browning during processing and storage. There is a lack of extensive research on the browning mechanism of wheat flour products using crude PPO extract as a variable, which was influenced by multiple factors. Indeed, many studies analyzed the mechanism of enzymatic browning by building crude PPO–substrate reaction systems or selecting different varieties of wheat flour as raw materials [2,3], which confirmed that wheat PPO plays an important role in the browning of wheat products. Therefore, the biochemical characterization of wheat PPO should be elucidated for the browning control of wheat products.

However, few researches have been published recently on the purification and characterization of wheat PPO from wheat. Bertrand and Muttermilch [4] first reported the PPO from wheat bran, and Anderson et al. [5] analyzed the biochemical and genetic characterization of wheat PPO. In contrast, PPO from fruits and vegetables has been extensively studied, including purification, kinetic parameters, substrate specificity, pH stability, thermal stability, and inhibition mechanisms. PPO from different sources possesses significantly different enzymatic properties, even among isoenzymes. It has been reported that the optimum pH of PPO is in the range of 5.0–8.0 and the optimum temperature is 20–50 °C [6,7,8]. A catechol substrate is the best substrate for purified PPO, while metal ions and inhibitors affect the enzyme differently depending on its source. Torres at al. [8] reported that Zn^2+^ could reduce the PPO activity of purple sweet potato while Cu^2+^ ions activated enzyme activity. Also, studies have shown that Cu^2+^ ions inhibit the activity of soluble PPO from *Prunus mume* but activate that of membrane PPO [9]. There has been evidence that ascorbic acid and sodium metabisulfite inhibit PPO in several plants, including green beans [10], fennel seeds [11]**,** mamey [12], and truffles [13]. Hence, the mutual reference information on different purified PPO properties is not applicable to a wide range of applications; therefore, research on the enzymatic properties of wheat PPO is essential for regulating wheat products’ browning or color changes.

The purpose of this study was to extract, purify, and analyze wheat bran PPO and analyze its molecular weight. Purified wheat PPO was investigated for substrate specificity, optimal pH and the stability of pH, optimal temperature and the stability of temperature, as well as the effects of metal ions and inhibitors. Based on the results of enzyme kinetics, four substrates including catechol, gallic acid, EGCG, and EC have been used for the molecular docking with PPO. These findings would provide a theoretical basis and an effective technical method for controlling the browning of wheat products and extending their shelf life based on the enzymatic properties of purified wheat PPO.

## 2. Results and Discussion

### 2.1. Purification and Molecular Weight of Wheat PPO

The purification stages and correlation results are shown in Table 1. The crude enzyme extracts from wheat bran were precipitated by (NH_4_)_2_SO_4_ precipitation. The precipitates were dissolved with buffer and dialyzed by the 10 kDa dialysis bags. Compared to crude enzyme extracts, the purified dialyzed enzyme’s yield and specific activity was 76.17% and 215.06 U/mg, respectively, with a purification efficiency of 1.74-fold. The dialyzed crude enzyme extracts were further purified using DEAE Sepharose Fast Flow exchange and Superdex G-75, resulting in purifications of 7.57-fold and 11.05-fold, respectively (Table 1). In the final purified wheat PPO, the overall activity yield and specific activity were 8.46% and 1365.12 U/mg, respectively. The same technology has been used to purify PPO from different fruits and vegetables. Ma et al. [9] purified the mPPO and sPPO from *Prunus mume* by performing a sequential purification using ammonium sulfate precipitation, dialysis, and DEAE Sepharose Fast Flow column steps. Their findings revealed that the mPPO and sPPO were purified 25.0-fold with a yield of 0.8% and 62.3-fold with a yield of 1.3%, respectively. Gong et al. [6] reported a 4.6-fold purification for PPO from the chestnut kernel with a yield of 2.03%.

**Figure 1:**
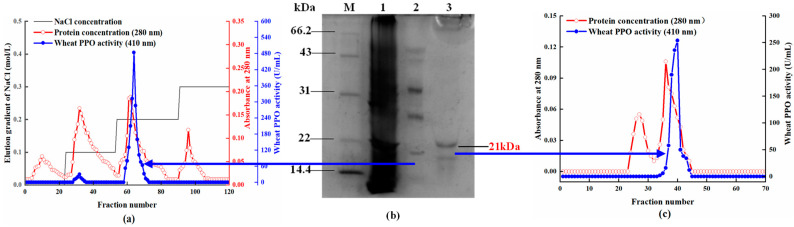
(**a**) DEAE-Sepharose fast flow column (absorbance at 280 nm and PPO activity) elution profiles of wheat bran polyphenol oxidase (PPO). (**b**) SDS-PAGE of different steps of wheat bran PPO purification. (Lane M: molecular weight markers; Lane 1: crude enzyme purified by (NH_4_)_2_SO_4_ precipitation; Lane 2: the enzyme purified by DEAE-Sepharose Fast Flow ion-exchange glass column; Lane 3: the enzyme purified by Superdex G-75 chromatography column). (**c**) Superdex G-75 gel filtration chromatography elution profile of wheat bran PPO.

Two protein peaks eluted from the DEAE Sepharose Fast Flow exchange column were measured as active, and the highest PPO activity was observed from recovered fractions of the second peak elution (Figure 1a). The purified enzyme solution in the second peak elution was collected, concentrated, and freeze-dried to be applied to the Superdex G-75 chromatography column. Figure 1c shows the elution profile of the highest active fractions, and a single protein peak with PPO active was obtained. The obtained active enzyme was used to compare the substrate specificity of the major phenolic compounds extracted from wheat flour and green tea. SDS-PAGE confirmed the wheat PPO partial purification, and the molecular weight of the obtained purified wheat PPO in this study was estimated to 21 kDa (Figure 1b). Compared to the multitude of different molecular weight proteins present in Lane 1 of the crude extract, the presence of multiple clear and scattered bands in Lane 2 is a confirmation of the effectiveness of this purification procedure. Superdex G-75 chromatography column was followed for further purification, revealing the presence of dual bands around 21 kDa and 17 kDa. PPO’s molecular weight from different sources has been reported to vary in the range of 14.2 kDa to 220 kDa [14,15]. These results might be due to species gene diversity and signal peptide cleavage [16].

### 2.2. Substrate Specificity and Enzyme Kinetics

There are various and abundant phenolic compounds in wheat flour and green tea that can contribute to enzymatic browning of raw materials and products during the processing and storage process. Therefore, wheat PPO was investigated for its ability to oxidize twelve diverse phenolic substrates from wheat grains and green tea. The highest relative activity was observed in the presence of catechol (100%) followed by gallic acid (89.20%), pyrogallic acid (86.64%), epigallocatechin gallate (83.69%), epicatechin (68.96%), epigallocatechin (67.19%), epicatechin gallate (51.87%), caffeic acid (29.08), protocatechuic acid (14.73%), phenol, vanillic acid, and ferulic acid (not examined) (Table 2). Consistent with our results, many PPO enzymes extracted from truffles [13], chestnut kernel [6], and plums [17] also showed the highest catechol activity. These findings suggested that the obtained, purified wheat PPO had substrate-binding sites with a high affinity for o-diphenolic (e.g., catechol and caffeic acid) and triphenolic (e.g., gallic acid and pyrogallic acid) structures. In contrast to previous reports, protocatechuic acid showed a lower affinity for wheat PPO, despite its o-diphenolic structure [18]. The phenolic compounds in green tea were more likely to become the substrate of wheat PPO because of their polyhydroxy structure, especially epigallocatechin gallate (EGCG). Numerous pieces of researches have shown that EGCG has a high affinity for PPO and inhibits its activity efficiently to prevent fruits and vegetable browning [19,20]. In addition, PPO from different plants has been applied to catalyze the oxidative polymerization of catechins to produce theaflavins and thearubigin [21,22].

According to the substrate specificity study, Kinetic constants (K_m_ and V_max_) were estimated using the Lineweaver–Burk plot. The used substrates, including catechol, gallic acid, pyrogallic acid, epigallocatechin gallate, epicatechin gallate, epicatechin, and epigallocatechin, measured the enzyme activity at various concentrations (2–10 mM) and under optimum analysis conditions. The K_m_ values are an index of the enzyme’s affinity towards its substrate. The purified wheat PPO exhibited the highest affinity for catechol (6.36 mM), gallic acid (7.05 mM), pyrogallic acid (7.37 mM), epigallocatechin gallate (7.70 mM), epicatechin gallate (7.90 mM), epicatechin (8.01 mM), and epigallocatechin (9.05 mM). The highest activity was observed with catechol (V_max_ 517.55 U·mL^−1^·min^−1^) followed by pyrogallic acid (V_max_ 500.84 U·mL^−1^·min^−1^), epigallocatechin gallate (V_max_ 455.17 U·mL^−1^·min^−1^), gallic acid (V_max_ 447.96 U·mL^−1^·min^−1^), epicatechin (V_max_ 417.15 U·mL^−1^·min^−1^), epicatechin gallate (V_max_ 400.43 U·mL^−1^·min^−1^), and epigallocatechin (V_max_ 319.44 U·mL^−1^·min^−1^). The results of this study are consistent with the reports of Wang et al. [23] and Lu et al. [17], which showed that the K_m_ and V_max_ values of PPO were 6.30 mM and 256.28 U/min for mango, and 10.32 mM and 64,520 U/(mL·min) for Chinese water chestnut based on catechol, respectively. In addition, wheat PPO has a high affinity for tea catechins, such as epigallocatechin gallate, epicatechin gallate, epicatechin, and epigallocatechin, which is reported for the first time in this study. Similar results have been reported by Ionita et al. [17]; the K_m_ and V_max_ of PPO from plums for catechin was 1.57 mM and 324.7 OD/min.

**Figure 2:**
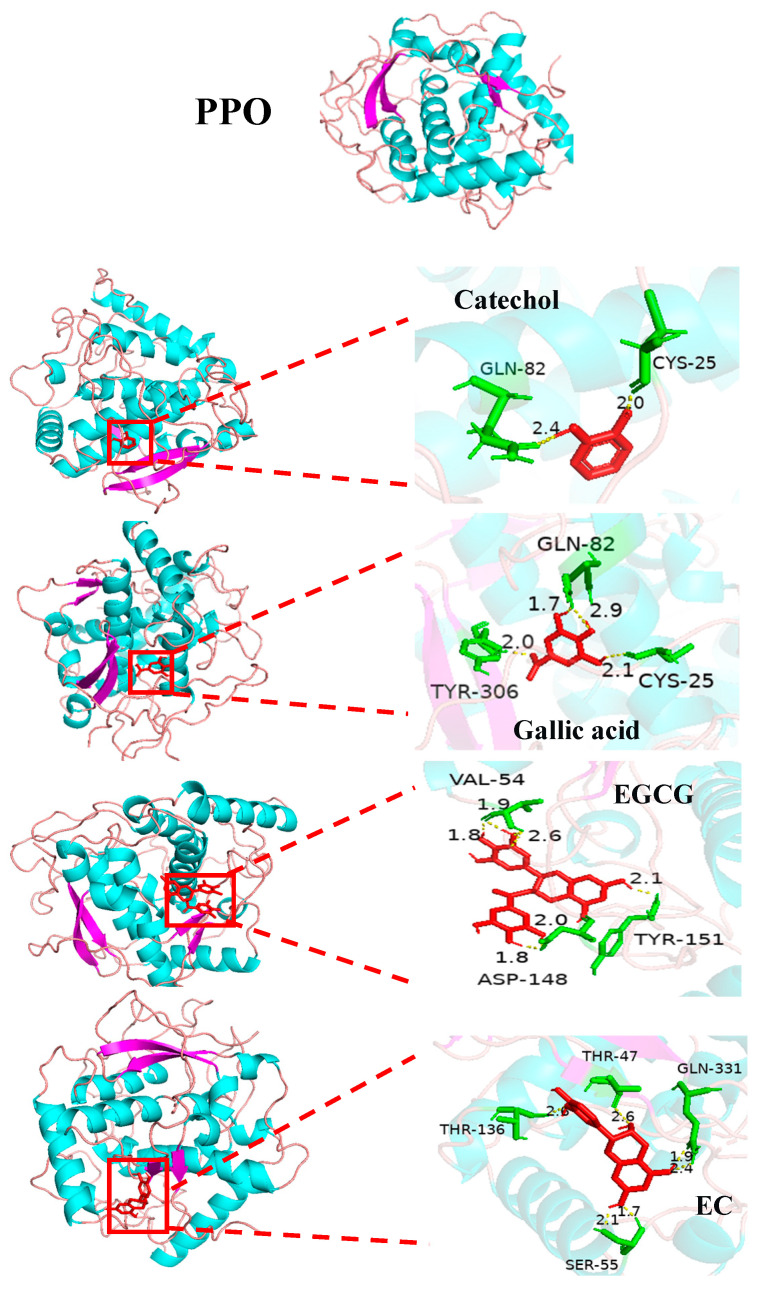
The molecular docking model of four substrates (catechol, gallic acid, EGCG, and EC) for binding of PPO.

To confirm the enzyme kinetics results and clarify the interaction mechanism, molecular docking was used for PPO (2P3X) and polyphenols by AutoDock (version 1.5.7). As shown in Figure 2, the binding energies of the four substrates with PPO were −5.28 kcal mol^−1^ (catechol), −4.65 kcal mol^−1^ (gallic acid), −4.21 kcal mol^−1^ (EGCG), and −5.62 kcal mol^−1^ (EC). In addition, catechol, gallic acid, EGCG, and EC with the amino acid residues of PPO formed 2, 4, 4, and 4 hydrogen bonds, respectively, and the hydrogen bond lengths were between 1.7 and 2.9 Å. These results suggested that the four polyphenol substrates all have higher affinity for PPO. It is worth noting that catechol, gallic acid, and EC completely entered the PPO cavity to interact with amino acid residues. Catechol and gallic acid formed hydrogen bonds with amino acid residues GLN 82 and CYS 52 of PPO due to their similar structure. While EGCG was combined with PPO, it entered only part of the structure into the interior of PPO, and the rest of the structure was free outside. These evidences strongly confirmed the results of substrate specificity and enzymatic kinetics.

### 2.3. Effect of pH on Wheat PPO Activity and Stability

**Figure 3:**
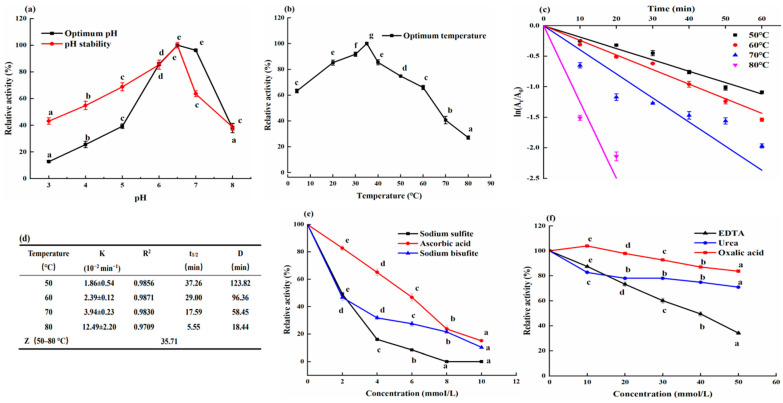
Partial enzymatic properties of purified wheat bran PPO. (**a**) Optimal pH and stability of purified wheat bran PPO. (**b**) Effect of temperature on the activity of purified wheat bran PPO. (**c**,**d**) Thermal inactivation of purified wheat bran PPO at different temperatures. (**e**,**f**) Effects of various inhibitors ((**e**) reducing agent; (**f**) chelating agent) on the activity of purified wheat bran PPO. Different lowercase letters show significant differences (*p* < 0.05).

pH changes greatly affect enzyme activity. Catechol was used as a substrate to determine the activity of purified wheat PPO in the pH range of 3.0–8.0. The purified wheat PPO presented the optimum activity at a pH of 6.5 (Figure 3a). Similar optimum pH was reported by Altunkaya and Gökmen [24], who measured the activity of durum wheat PPO in the pH range of 4.0–9.0 with an optimum pH of 6.5. Benaceur et al. [13] reported an optimum pH of 7.0 for PPO from truffles, Siddiq and Dolan [25] reported an optimum pH of 6.1 for PPO from blueberry, and PPO from borage showed optimum activity at pH 7.5 [26]. In the pH range of 3.0–6.5, purified wheat PPO activity increased slowly, followed by a sudden decrease in the pH range of 6.5–8.0 (Figure 3a). These phenomena showed that purified wheat PPO was more sensitive to alkaline environment. Nevertheless, the relative activity of purified wheat PPO has been as low as 12.78% at pH 3.0.

As shown in Figure 3a, the pH stability of purified wheat PPO was evaluated at 4 °C for 24 h by incubating the enzyme in a pH range of 3.0–8.0. The relative activity value of purified wheat PPO expressed optimal stability at pH 6.5. When pH 6.5 and 7.0 were used, purified wheat PPO retained greater than 90% relative activity, whereas at pH 8.0 it retained 38.63% relative activity. It was observed that the relative activity of purified wheat PPO decreased with the decreasing pH below 6.5. At pH 3.0, the relative activity of purified wheat PPO lost more than 50% of its initial activity. In contrast, purified wheat PPO exhibited better stability under acidic conditions, and these results are accordant with the research results of others [14,17].

### 2.4. Effect of Temperature on Wheat PPO Activity and Stability

A study was conducted to determine the optimal temperature and activity stability of purified wheat PPO under different temperatures in the range of 4 °C to 80 °C. The purified wheat PPO showed the highest activity at an incubation temperature of 35 °C (Figure 3b), which was similar to the results reported by Siddiq and Dolan [25] who found that the optimal temperature of blueberry PPO was 35 °C. The optimal temperature for wheat PPO was also reported by other studies to display at 35 °C and 40 °C [24,27]. The activities of purified wheat PPO at temperatures ranging from 4 to 60 °C were always above 60%, as shown in Figure 3b. The enzyme activity decreased sharply as the temperature increased above 65 °C, but the enzyme activity was not completely destroyed even at 80 °C, which was consistent with that in the temperature properties of PPO from truffles [13].

An incubation at 50, 60, 70, and 80 °C for 1 h was used to evaluate the thermal stability of purified wheat PPO. When the temperature is below 70 °C, approximately 60 min would be taken to inactivate 90% of the purified wheat PPO (Figure 3c,d). The same phenomena have been observed for PPO from other plants. Nagai and Suzuki [28] reported that PPO from Chinese cabbage was stable in the range of 50 to 70 °C but inactivated above 70 °C. By comparison, the PPO from mamey was inactivated above 65 °C [12]. When the incubation temperature reached 80 °C, 90% of the purified wheat PPO was inactivated within 18.44 min. The K values increased from 1.86 × 10^−2^ to 12.49 × 10^−2^ min^−1^ with the increasing incubation temperature from 50 to 80 °C, confirming purified wheat PPO’s thermal sensitivity. The Z value is commonly used to evaluate the thermal sensitivity parameter of enzymes. In this study, the Z value of purified wheat PPO was 35.71 °C (Figure 3d). Comparing the Z values of purified wheat PPO with other PPOs utilizing catechol as substrate, we discovered that purified wheat PPO was more thermostable than durum wheat PPO (23.4 °C) [24] and plum PPO (15.15 °C) [17] but proximal to myrtle PPO (33.40 °C) [29] and amorphophallus PPO (34.01 °C) [30].

### 2.5. Effect of Metal Ions on Wheat PPO Activity

Using catechol as a substrate, six metal ions were tested on purified wheat PPO at two different inhibitor concentrations (1 mM and 10 mM). As seen in Table 3, the effect of metal ions on the activity of purified wheat PPO has both activation and inhibition behavior. It was found that monovalent metal ions (K^+^ and Na^+^) inhibited the activity of purified wheat PPO compared to divalent metal ions (Mg^2+^, Ca^2+^, Cu^2+^). In contrast, the inhibitions of the two ions were not markedly different at concentrations of 1 mM and 10 mM. Cu^2+^ at 1 mM and 10 mM presented the most significant activation, with a 3.79% and 48.79% increase in the initial activity, respectively. The results are similar to those reported for truffle PPO [13] and green bean PPO [10] while inconsistent with the results reported by Gao et al. [7] for red Swiss chard leave PPO. Mg^2+^ at 1 mM exerted an inhibitory effect with a 19.09% loss of initial activity, while Mg^2+^ at 10 mM reacted as activators with a 0.45% increase in initial activity. The findings of other authors were also similar to those of Ma et al. [9], who found that Mg^2+^ at 0.05 mol/L inhibited the relative activity of mPPO from Prunus mume but at 0.1 mol/L and 0.15 mol/L increased the relative activity of mPPO. The highest inhibitory effect was observed with Ca^2+^ at 10 mM, which inhibited 45.76% of the enzyme activity. In brief, the stimulatory effect of metal ions (Mg^2+^, Cu^2+^) can be explained by increased enzyme stability and the affinity between enzyme and substrate. In contrast, some metal ions (Ca^2+^, Na^+^, K^+^) disrupt the enzyme activity by changing the conformation of the enzyme through chemical bonds [9,11].

### 2.6. Effect of Inhibitors on Wheat PPO Activity

Figure 3e,f shows the effects of six different inhibitors on purified wheat PPO activity, determined using catechol as substrate. From 2 to 10 mM of sodium sulfite, ascorbic acid, and sodium bisulfite, purified wheat PPO activity was strongly inhibited (Figure 3e). Sodium sulfite, at a concentration of 8 mM, inhibited 100% of the purified wheat PPO activity, while ascorbic acid and sodium bisulfite, at a concentration of 10 mM, inhibited 85.88% and 89.72% of the purified wheat PPO activity, respectively. These results are in good agreement with what was reported by Gao et al. [7] and Ionita et al. [17]. However, EDTA, urea, and oxalic acid had a weak impact on purified wheat PPO activity. EDTA at 50 mM only exhibited 65.82% inhibition. Ionita et al. [17] also reported a lower inhibition effect for EDTA at 50 mM with a 20.75% inhibition degree. As a result of a 50 mM concentration, the lowest inhibition effects were observed with urea and oxalic acid at 29.07% and 16.31% inhibition, respectively. Conversely, a stronger inhibition effect was observed for urea at 5 mM with 100% inhibition from the inhibition studies with African bush mango fruit peel PPO [16]. Oxalic acid at 5 mM was defined as a medium effective inhibitor for fennel seed PPO with 41% inhibition [11], which had a stronger inhibitory effect than in the inhibition results of this study. The inhibition effect is due to its ability to change the pH of the reaction system, chelate the copper ion in the PPO active center, reduce the intermediates caused by enzyme browning, and transform light or colorless substances from the intermediates by combination. Based on this, reducing agents (sodium sulfite, ascorbic acid, and sodium bisulfite) presented a certain inhibitory effect on PPO from different sources [17,25], which have been extensively reported. Compared to the chelating agent and acidifier, the reducing agent significantly inhibited the purified wheat PPO activity at low doses.

## 3. Materials and Methods

### 3.1. Materials and Chemical Reagents

Wheat bran (*Wan grano*) was purchased from a peasant household in Wuhe County, Bengbu, Anhui Province, China. All of the analytical pure chemicals were obtained from Sinopharm chemical reagent Co., Ltd. (Shanghai, China). Standard reagents of HPLC grade were bought from Shanghai Yuanye Biotechnology Co., Ltd. (Shanghai, China).

### 3.2. Extraction and Purification of Wheat PPO

Crude enzyme extractions were carried out following the method of Zhou et al. [14] with minor modifications. Powdered wheat bran (15 g) was homogenized on the stir for 8 h at 4 °C in 150 mL of 50 mM phosphate buffer (pH 6.5) containing 1% polyvinylpolypyrrolidone (PVPP). The homogenate was centrifuged at 10,000× *g* for 0.5 h at 4 °C. To obtain 30% saturation at 0 °C, ammonium sulfate was stirred into the supernatant enzyme solution. Then, the mixture solution was centrifuged at 10,000× *g* for 0.5 h at 4 °C after resting 2 h. The obtained supernatant was again treated with ammonium sulfate by stirring to attain 80% saturation at 0 °C, and the previous resting and centrifugal process was repeated. The precipitate was collected and dissolved into 50 mM phosphate buffer (pH 6.5). The crude enzyme solution was dialyzed in 10 kDa dialysis bags at 4 °C against the same buffer solution for 48 h with eight buffer solution changes. BaCl_2_ was used to determine the presence of ammonium sulfate until no precipitation generated in the dialysate.

The dialyzed crude enzyme solution was fractionated by DEAE-Sepharose Fast Flow ion-exchange glass column (16 mm × 400 mm) (Shanghai Xiamei Biochemical Technology Development Co., Ltd., Shanghai, China). The column was preequilibrated with 20 mM Tris-HCl buffer (pH 8.0). The dialyzed crude enzyme solution was passed through the column and washed with the same Tris-HCl buffer. The column was eluted with a linear gradient of NaCl concentration from 0.10 to 0.3 mol/L in Tris-HCl buffer (pH 8.0) at a 1.2 mL/min flow rate. Fractions of 6 mL were collected to analyze wheat PPO activities, and their absorbance was determined at 280 nm. The fractions of high wheat PPO activity were collected.

The Superdex G-75 chromatography column (16 mm × 600 mm) (Beijing Ruida Henghui Technology Development Co., Ltd., Beijing, China) was then applied to further purify wheat PPO. The column was washed and equilibrated with phosphate buffer (50 mM pH 7.0) before use. Freeze-dried enzyme powder was dissolved in the same buffer; the dissolved enzyme solution was passed through the Superdex G-75. The column was eluted with the same buffer at the flow rate of 0.3 mL/min, and fractions of 3 mL were collected every 10 min. Enzyme activity and protein content were determined during purification. The high-PPO active components of wheat were collected and concentrated with an ultrafiltration membrane (10 kDa), freeze-dried, and preserved at −18 °C for analysis.

### 3.3. Wheat PPO Assays and Protein Concentration

A spectrophotometric method was used to assess wheat PPO activity by measuring the increase in absorbance at 410 nm using catechol [17]. Briefly, the sample contained 100 µL of the enzyme solution and 1.9 mL of 10 mM catechol solution prepared with 50 mM phosphate buffer (pH 6.5). The reference sample contained 1.9 mL of catechol solution with 0.1 mL 50 mM phosphate buffer (pH 6.5). The sample and blank reaction solutions were kept at 35 °C for 5 min. One unit of wheat PPO activity is defined as a change in solution absorbance of 0.001 per minute after the reaction.

The method of the wheat PPO extracts’ protein concentration was measured referring to the report of Torres et al. [8]. Briefly, the sample mixture contained diluted tenfold enzyme and 5 mL Bradford reagent. Various volumes of bovine serum protein (1 mg/mL) (0, 0.2, 0.4, 0.6, 0.8, and 1.0 mL) were diluted to 1 mL with distilled water; then, the Bradford reagent was added (5 mL). All the reaction mixtures reacted for 5 min at room temperature, and the absorbance was measured at 595 nm. The obtained data from the bovine serum albumin as a protein standard were drawn from the standard curves. The protein concentration of wheat PPO was calculated using this standard graph.

### 3.4. Estimation of Molecular Weight

The molecular weight of purified wheat PPO was estimated by SDS-PAGE according to the method of Karakus et al. [11]. The purified PPO and Markers were mixed with the sample loading buffer at a ratio of 4:1 and incubated in boiling water for 5 min. Then, the mixture was centrifuged at 10,000× *g* for 5 min at room temperature. A total 10 µL 0.2 mg/mL of the supernatant and markers were injected in the gel. Electrophoresis was run at 100 V at room temperature until the bromophenol blue marker was observed at the bottom. The stacking and separating gels were prepared with 5.0% and 12% acrylamide content, respectively. After electrophoresis, the gel was stained with 2.5 g/L of Coomassie Brilliant Blue G-250 for 0.5~1 h. The gels were decolorized until the bands were clear. The standard molecular weight markers (Beijing Solarbio Science and Technology Co., Ltd., Beijing, China) were used for estimating molecular weights of purified wheat PPO.

### 3.5. Substrate Specificity and Kinetic Parameters K_m_ and V_max_

The substrate specificity research was performed with twelve different substrates, including phenol, catechol, ferulic acid, protocatechuic acid, vanillic acid, caffeic acid, gallic acid, pyrogallic acid, epicatechin, epicatechin gallate, epigallocatechin, and epigallocatechin gallate at a concentration of 10 mM. A 50 mM phosphate buffer solution (pH 6.5) was used to prepare the substrate solutions. We mixed 1.9 mL of different substrate solutions with 0.1 mL of 0.25 mg/L purified wheat PPO solution separately and measured enzyme activity according to the unified standard method of 2.3.

The maximum reaction rate (V_max_) and Michaelis–Menten constant (K_m_) of 0.25 mg/L purified wheat PPO were assayed using the method by varying the concentrations of catechol (2, 4, 6, 8, and 10 mM) in a 50 mM phosphate buffer solution (pH 6.5). The obtained data were plotted using the Michaelis–Menten equation and Lineweaver–Burk plot, referring to the Ma method [9].

### 3.6. pH Optimum and Stability

The optimum pH for purified wheat PPO activity was determined by monitoring the various pH values (3.0~8.0), and catechol was selected as a substrate. The pH stability of 0.25 mg/L purified wheat PPO activity was measured by incubating wheat PPO in 50 mM phosphate buffer (pH 3.0~8.0) for 24 h at 4 °C. Wheat PPO activity was analyzed at the end of culture according to the unified standard method of Section 3.3.

### 3.7. Thermal Activity and Stability

The optimum temperature of 0.25 mg/L purified wheat PPO was determined in the range of 4~80 °C at pH 6.5. The substrate solution (10 mM pH 6.5 catechol solution) was pre-heated at different temperatures for 5 min. The wheat PPO solution was added to the substrate and incubated at the corresponding temperature for 5 min. After the reaction, the residual activity of PPO was determined according to the unified standard method of Section 3.3.

Thermal stability of 0.25 mg/L purified wheat PPO was determined by dissolving it in 50 mM pH 6.5 phosphate buffer and incubating it for 60 min at different temperatures (50, 60, 70, and 80 °C). During the cultivation process, approximately 0.6 mL of enzyme solution was taken out for analyzing the enzyme activity every 10 min.

The thermal inactivation kinetics of PPO was based on the first-order kinetic reaction (Equation (1)):

(1) ln ⁡A0∕At=−kt

where A_0_ and A_t_ are shown as the original activity of the samples and residual activity of the samples after heat treatment, respectively, and k is the inactivation rate constant (min^−1^).

The half-life ($t_{\frac{1}{2}}$) value of inactivation is expressed as Equation (2):

(2) $$t_{\frac{1}{2}}=\ln\left(2\right)/k$$

The time required for a 90% decline in original enzyme activity is defined as Equation (3):

(3) D=ln ⁡10/k

The Z value is the temperature when the D value decreases by a logarithmic period, which is acquired by plotting the relationship between the log D value and the temperature.

### 3.8. Effect of Inhibitors and Metal Ions on Wheat PPO Activity

Six inhibitor effects (ascorbic acid, sodium sulfite, sodium bisulfite, urea, oxalic acid, and EDTA) on wheat PPO activity were investigated at different concentrations. The effect of metal ions (Na^+^, K^+^, Mg^2+^, Ca^2+^, and Cu^2+^) at final concentrations (1 mM and 10 mM) on the enzyme activity was analyzed. The reaction mixture containing 1.9 mL of catechol prepared with ultrapure water, 0.1 mL of inhibitor solution or metal ion solution, and 0.1 mL of 0.25 mg/L purified wheat PPO was incubated at 35 °C. After reacting for 5 min, the residual enzyme activity was analyzed.

### 3.9. Molecular Docking

The interaction between PPO and four substrates (catechol, gallic acid, EGCG, and EC) was simulated by molecular docking, referring to the method of Tian et al. [19]. AutoDock (version 1.5.7) was used for PPO-polyphenol substrate docking. The crystal structure of PPO (2P3X) was obtained from the database (https://www.rcsb.org/pdb, accessed on 8 October 2023), and the molecule structure of the four substrates was obtained from PubChem (https://pubchem.ncbi.nlm.nih.gov/, accessed on 8 October 2023). The following parameters were used for molecular docking: center x = 25.3, center y = −8.0, and center z = 17.1; search space—size x: 60, size y: 60, size z: 60 (the distance between each grid point was 0.375Å), exhaustiveness: 10, and the remaining parameters were default settings. PyMOL (version 2.6.0a0) was used for analysis and the optimal docking conformation was chosen based on the lowest binding energy.

### 3.10. Statistical Analysis

The results were presented as mean values of triplicate measurements with standard deviations (mean ± SD, *n* ≥ 3). Statistical analyses were performed using SPSS 23 software (SPSS Inc., Chicago, IL, USA). The statistical significance was processed by LSD post hoc test, and the level of significance was set at *p* < 0.05.

## 4. Conclusions

For the first time, wheat PPO was purified and biochemically characterized from wheat bran (*Wan grano*, produced in Bengbu, Anhui). Wheat PPO was extracted and purified through ammonium sulfate precipitation, DEAE-Sepharose Fast Flow ion-exchange glass column, and Superdex G-75 chromatography column. With an overall activity yield of 8.46%, the final purification step achieved an 11.05-fold purification. SDS-PAGE results indicated that the presence of purified wheat PPO exhibited a molecular weight of 21 kDa. The highest catalytic efficiency was observed for catechol as substrate, followed by gallic acid, pyrogallic acid, epigallocatechin gallate, epicatechin, epigallocatechin, and epicatechin gallate. According to the enzyme kinetics parameters, the purified wheat PPO showed variable affinity for different substrates. Catechol was the most preferred substrate due to its maximum V_max_ (517.55 U·mL^−1^·min^−1^) and low K_m_ (6.36 mM) values. The optimal conditions for enzyme activity were a temperature of 35 °C and a pH of 6.5. The purified wheat PPO presented higher stability in the pH range of 6.0–7.0 and temperature domain of 20–60 °C. Results from thermal inactivation of purified wheat PPO at 50–80 °C evidenced K values varying from 1.86 × 10^−2^ to 12.49 × 10^−2^ min^−1^ and D-values between 123.82 and 18.44 min. The purified wheat PPO was sensitive to some general PPO inhibitors, especially sodium sulfite, ascorbic acid, and sodium bisulfite. The metal ions have double-sided effects on the purified wheat PPO; for example, Cu^2+^ significantly activated the enzyme activity, while Ca^2+^ significantly inhibited the enzyme activity. A comprehensive understanding of the biochemical properties of PPO isolated from local wheat bran will establish a theoretical foundation for inhibiting enzymatic browning reactions and better technical services for local wheat product processors.

## Figures and Tables

**Table 1:** Isolation and purification of PPO from wheat bran.

Purification Step	Total Protein Content (mg)	Total Activity (U)	Specific Activity (U/mg)	Purification Fold	Yield (%)
Crude extract	207.03 ± 2.91	25,600 ± 1705.87	123.59 ± 6.48	1	100.00
(NH_4_)_2_SO_4_ precipitation (80%)	90.60 ± 0.58	19,500 ± 793.73	215.06 ± 7.92	1.74	76.17
DEAE Sepharose Fast Flow	4.13 ± 0.04	3870 ± 108.17	936.18 ± 16.69	7.57	15.12
Superdex G-75	1.59 ± 0.05	2165 ± 22.91	1365.12 ± 28.80	11.05	8.46

**Table 2:** Substrate specificity and kinetics parameters of purified wheat bran PPO.

Substrate (Concentration = 10 mM)	Relative Activity (%)	K_m_ (mM)	V_max_ (U·mL^−1^·min^−1^)	V_max_/K_m_ (U·mL^−1^·min^−1^·mM^−1^)
phenol	0 ^a^	-	-	-
catechol	100.00 ± 0.80 ^g^	6.36 ± 0.71	517.55 ± 15.20	81.29
ferulic acid	0 ^a^	-	-	-
vanillic acid	0 ^a^	-	-	-
caffeic acid	29.08 ± 0.68 ^c^	-	-	-
protocatechuic acid	14.73 ± 3.58 ^b^	-	-	-
gallic acid	89.20 ± 0.68 ^f^	7.05 ± 0.47	447.96 ± 11.41	63.54
pyrogallic acid	86.64 ± 2.13 ^f^	7.37 ± 0.71	500.84 ± 25.08	67.96
epicatechin	68.96 ± 2.92 ^e^	8.01 ± 0.46	417.15 ± 17.39	52.08
epicatechin gallate	51.87 ± 3.51 ^d^	7.90 ± 0.64	400.43 ± 17.39	50.69
epigallocatechin	67.19 ± 0.97 ^e^	9.05 ± 0.18	319.44 ± 16.03	35.30
epigallocatechin gallate	83.69 ± 2.41 ^f^	7.70 ± 0.14	455.17 ± 20.71	59.11

Different lowercase letters show significant differences (*p* < 0.05).

**Table 3:** Effect of metal ions on the activity of purified wheat bran PPO.

Metal Ions	Relative Activity (%)	
	1 mM	10 mM
blank	100	100
Na^+^	84.09 ± 1.98 ^b^	82.88 ± 1.46 ^b^
K^+^	86.06 ± 3.03 ^b^	81.66 ± 2.77 ^b^
Mg^2+^	80.91 ± 3.18 ^b^	100.45 ± 2.00 ^c^
Ca^2+^	74.55 ± 4.39 ^a^	54.24 ± 1.60 ^a^
Cu^2+^	103.79 ± 1.14 ^c^	148.79 ± 2.05 ^d^

Different lowercase letters show significant differences (*p* < 0.05).

## Data Availability

All data are included in the manuscript, and all results of this study are available from the corresponding author.
